# Assessment of Cultural Beliefs and Practices During the Postnatal Period Affecting Postpartum Recovery and Its Management in a Tertiary Care Hospital

**DOI:** 10.7759/cureus.114259

**Published:** 2026-08-10

**Authors:** Mahendra Gangadharaiah, Tejaswini M Mudri

**Affiliations:** 1 Obstetrics and Gynecology, Adichunchanagiri Institute of Medical Sciences, B.G. Nagara, IND

**Keywords:** cultural beliefs, dietary practices, food taboos, postnatal women, postpartum period

## Abstract

Introduction and aim: The postpartum period is associated with several physiological and nutritional changes, during which women commonly follow culturally influenced dietary practices and traditional beliefs. These practices may influence maternal nutrition, physical activity, and postpartum recovery. The present study aimed to assess cultural beliefs, dietary practices, lifestyle-related factors, myths, and traditional postpartum practices among postnatal women attending a tertiary care hospital, and to examine their association with self-reported postpartum weight change.

Materials and methods: This prospective observational study was conducted among 100 postnatal women attending the Department of Obstetrics and Gynecology at Adichunchanagiri Institute of Medical Sciences, Karnataka, India, from January 2026 to April 2026. Data were collected using a predesigned semi-structured questionnaire developed after an extensive review of published literature and previously validated studies on postpartum cultural practices. The questionnaire underwent content validation by experts in obstetrics and community medicine and was pretested among 10 postnatal women to ensure clarity and feasibility before administration. It assessed sociodemographic characteristics, dietary practices, cultural beliefs, lifestyle modifications, preferred and restricted foods, and self-reported postpartum weight change. Data were analyzed using SPSS version 26.0 (Armonk, NY: IBM Corp.).

Results: Restriction in quantity of food intake was practiced by 97 (97.0%) women, while consumption of “hot foods” was reported by 87 (87.0%) participants. Reduced water intake was observed among 57 (57.0%) women. Head covering, confinement at home, and reduced physical activity were practiced by 92 (92.0%), 86 (86.0%), and 81 (81.0%) women, respectively. Restriction of curd and buttermilk was observed among 32 (32.0%) participants, whereas restriction of protein-rich foods was reported by eight (8.0%) women. Weight loss during the postpartum period was reported by 46 (46.0%) participants. Consumption of “hot foods” demonstrated a significant association with self-reported postpartum weight change (χ²=9.097, p=0.011), whereas no significant associations were observed for parity, mode of delivery, religion, restriction in quantity of food intake, or vegetable restriction (p>0.05).

Conclusion: Traditional postpartum dietary practices and cultural beliefs were highly prevalent among postnatal women. Several culturally accepted food restrictions and lifestyle practices continued to influence postpartum dietary habits and were associated with self-reported postpartum weight change. However, given the cross-sectional design and reliance on self-reported outcome measures, these findings should be interpreted with caution. Further multicenter longitudinal studies with objective nutritional assessment are warranted.

## Introduction

The postpartum period is a critical phase in a woman’s life characterized by physiological, psychological, and social adjustments following childbirth [1]. Appropriate nutrition, hydration, physical activity, and emotional support during this period play a vital role in maternal recovery, lactation, and overall well-being [2]. However, postpartum practices are often strongly influenced by cultural beliefs, traditional customs, and family advice, particularly in developing countries such as India [3].

Across different communities, several dietary restrictions and traditional postpartum practices are commonly followed with the belief that they promote healing, improve lactation, prevent infections, and restore maternal strength [4]. Foods considered “cold,” such as curd, buttermilk, fruits, and certain vegetables, are frequently avoided, while “hot foods” and herbal preparations are encouraged [5,6]. Practices such as confinement at home, reduced physical activity, head covering, abdominal binding, and fluid restriction are also commonly observed during the puerperal period [7]. Although many of these practices are culturally accepted and transmitted across generations, some may adversely affect maternal nutritional status and postpartum recovery [8].

Postpartum food taboos and myths may lead to inadequate dietary diversity, reduced fluid intake, micronutrient deficiencies, constipation, fatigue, delayed wound healing, and impaired recovery [9]. At the same time, certain traditional practices may provide psychological comfort and social support to the mother [10]. Understanding these culturally rooted beliefs and practices is important for healthcare professionals in order to provide culturally sensitive and evidence-based counseling and promote healthy postpartum behaviors without disregarding traditional values [11].

Despite the widespread prevalence of postpartum cultural practices in India, limited studies have evaluated their influence on dietary habits, lifestyle modifications, and self-reported postpartum weight change among postnatal women attending tertiary care hospitals [12,13].

## Materials and methods

This prospective observational study was conducted in the Department of Obstetrics and Gynecology at Adichunchanagiri Institute of Medical Sciences, Adichunchanagiri University, Karnataka, India, among postnatal women attending the obstetrics and postnatal services from January 2026 to April 2026 after obtaining approval from the Institutional Ethics Committee (#AIMS/IEC/028/2025). The study was undertaken to assess cultural beliefs, dietary practices, lifestyle-related factors, myths, and traditional postpartum practices affecting postpartum recovery during the postnatal period. Written informed consent was obtained from all study participants before data collection. Confidentiality and anonymity of the participants were maintained throughout the study.

This study included postnatal women within six weeks following delivery who were willing to participate. Women who were critically ill, unable to respond appropriately, or unwilling to provide informed consent were excluded from the study. Eligible participants were recruited using convenience sampling until the required sample size was achieved. The sample size was calculated using the formula below based on the findings of Venkateswarlu et al., wherein 79.6% of postnatal women reportedly did not go out for walks during the postpartum period [6].

$n = \frac{4pq}{d^2}$

Here, p=79.6%, q=20.4%, and the allowable error (d) was taken as 10% of p (7.96); the calculated sample size was approximately 64. To improve the precision of estimates and to account for possible incomplete questionnaires or missing data, a total of 100 postnatal women were enrolled in this study.

Data were collected using a predesigned semi-structured questionnaire specifically developed for the present study after an extensive review of published literature on postpartum cultural beliefs and practices and previously published questionnaires assessing postpartum dietary and lifestyle behaviors. The initial questionnaire was prepared by the investigators and reviewed independently by three subject experts (two obstetricians and one community medicine specialist) to assess content relevance, clarity, comprehensiveness, and appropriateness. Based on their suggestions, minor modifications in wording and sequencing of questions were made to improve readability.

The revised questionnaire was subsequently pre-tested among 10 postnatal women who fulfilled the eligibility criteria but were not included in the final analysis. The pre-testing evaluated question clarity, ease of understanding, language, and administration time. Minor modifications were incorporated following participant feedback. The questionnaire was administered by the investigators through face-to-face interviews using a standardized approach to minimize interviewer-related variability. The final questionnaire consisted of sections assessing sociodemographic characteristics, obstetric history, dietary practices, food restrictions, culturally perceived beneficial foods, fluid intake, physical activity, confinement practices, traditional postpartum customs, and self-reported postpartum weight change. The complete questionnaire is provided in the appendix.

Self-reported postpartum weight change was assessed based on participants’ perception of body weight change during the postpartum period compared with their immediate post-delivery status and categorized as weight loss, weight gain, or no significant change. Weight loss was defined as a reduction in body weight compared to immediate post-delivery weight, while weight gain referred to an increase in body weight during the postpartum period. No significant change was defined as variation within ±1 kg of the immediate post-delivery weight. As this outcome was based on participant self-report rather than direct measurement, it was interpreted as a subjective indicator of perceived postpartum weight change. “Hot foods” referred to culturally perceived heat-producing foods such as pepper, garlic, onion, ginger, and traditional herbal preparations believed to improve lactation and postpartum recovery.

Data were entered into Microsoft Excel 2021 (Redmond, WA: Microsoft Corp.) and analyzed using IBM Statistical Package for the Social Sciences version 26.0 (Armonk, NY: IBM Corp.) for Windows. Categorical variables were summarized as frequency and percentage. Associations between categorical variables were evaluated using Pearson’s chi-square test when all expected cell frequencies were ≥5. Fisher’s exact test was used whenever expected cell frequencies were <5. All statistical tests were two-tailed, and p<0.05 was considered statistically significant.

## Results

Among the 100 postnatal women included in the study, the majority belonged to the Hindu (94, 94.0%), while Muslims constituted six (6.0%) of the study population. Primiparous women accounted for 53 (53.0%) participants and multiparous women for 47 (47.0%). Regarding mode of delivery, 55 (55.0%) women underwent lower segment cesarean section (LSCS) and 45 (45.0%) had vaginal delivery (Table 1).

**Table 1:** Sociodemographic and obstetric characteristics of study participants (n=100). Data are presented as n (%). The table summarizes the baseline sociodemographic and obstetric characteristics of the study participants, including religion, parity, and mode of delivery. LSCS: lower segment cesarean section

Variables	Category	n (%)
Religion	Hindu	94 (94)
	Muslim	6 (6)
Parity	Primipara	53 (53)
	Multipara	47 (47)
Mode of delivery	LSCS	55 (55)
	Vaginal delivery	45 (45)

Dietary restrictions and culturally influenced food practices were highly prevalent during the postpartum period. Restriction in quantity of food intake was practiced by 97 (97.0%) women. Consumption of culturally perceived “hot foods” such as pepper-, garlic-, and onion-based preparations was reported by 87 (87.0%) participants. Restriction of curd and buttermilk was observed among 32 (32.0%) women, while restriction of brinjal, potato, and pumpkin was reported by 18 (18.0%). Reduced water intake of less than 2 L/day was noted in 57 (57.0%) women. Nearly all participants consumed toor daal (Hindi) with rice (99, 99.0%) during the postpartum period (Table 2).

**Table 2:** Dietary beliefs and cultural food practices during the postnatal period (n=100). Data are presented as n (%). The table describes the frequency of dietary beliefs and cultural food practices followed by participants during the postpartum period, including food restrictions, consumption of culturally preferred foods, and water intake practices.

Dietary practice/belief	n (%)
Restriction in quantity of food intake	97 (97)
Consumption of “hot foods”	87 (87)
Consumption of vegetables	98 (98)
Restriction of curd/buttermilk	32 (32)
Restriction of brinjal/potato/pumpkin	18 (18)
Restriction of fruits	6 (6)
Restriction of protein-rich foods (eggs/fish/mutton)	8 (8)
Reduced water intake (<2 L/day)	57 (57)
Consumption of toor daal (Hindi) with rice	99 (99)

Traditional postpartum practices and lifestyle modifications were commonly followed among the participants. Head covering was practiced by 92 (92.0%) women, while restriction of outdoor activities and confinement at home were observed in 86 (86.0%) women. Reduced physical activity was reported by 81 (81.0%) participants. Oil massage/body massage practices and use of home remedies or Ayurvedic preparations were reported by 74 (74.0%) and 73 (73.0%) women, respectively. Abdominal binding was practiced by 56 (56.0%) participants, whereas warm clothing irrespective of climate was followed by 22 (22.0%) women (Table 3).

**Table 3:** Traditional postpartum cultural practices and lifestyle modifications (n=100). Data are presented as n (%). The table summarizes traditional postpartum cultural practices and lifestyle modifications adopted by the participants, including head covering, confinement, physical activity, massage, abdominal binding, and use of home remedies.

Practice	n (%)
Head covering	92 (92)
Restriction of outdoor activity/confinement at home	86 (86)
Reduced physical activity	81 (81)
Oil massage/body massage	74 (74)
Consumption of home remedies/Ayurvedic preparations	73 (73)
Abdominal binding	56 (56)
Warm clothing irrespective of climate	22 (22)

Several foods were culturally restricted during the postpartum period because of prevailing myths and traditional beliefs. Restriction of curd and buttermilk was reported by 32 (32.0%) women due to the belief that these foods are “cold” and may delay recovery or cause cough and cold. Restriction of brinjal, potato, and pumpkin was observed among 18 (18.0%) participants because these foods were believed to predispose to infection, itching, abdominal distension, and indigestion. Restriction of eggs, fish, and mutton was reported by eight (8.0%) women owing to beliefs regarding impaired digestion and delayed wound healing. Reduced water intake was practiced by 57 (57.0%) women because of beliefs related to abdominal distension, back strain, and increased urination (Table 4).

**Table 4:** Commonly restricted foods during the postnatal period and associated cultural beliefs (n=100). Data are presented as n (%). The table outlines the commonly restricted foods and dietary practices during the postpartum period along with the corresponding cultural beliefs or perceived reasons for these restrictions.

Restricted food item/practice	n (%)	Common cultural belief/reason
Curd and buttermilk	32 (32)	Considered “cold foods”; believed to delay recovery and cause cold/cough
Brinjal	18 (18)	Believed to predispose to infection “nanju” and itching
Potato and pumpkin	18 (18)	Believed to cause abdominal distension and indigestion
Fruits (banana/apple/guava)	6 (6)	Believed to cause cold and cough in mother and infant
Eggs, fish, and mutton	8 (8)	Believed to impair digestion and delay wound healing
Sesame seeds	8 (8)	Believed to increase body heat
Reduced water intake	57 (57)	Believed to cause abdominal distension, increased urination, and back strain

Preferred foods during the postpartum period mainly included culturally perceived recovery-promoting preparations. Consumption of “hot foods” such as pepper-, garlic-, and onion-based preparations was reported by 87 (87.0%) women because they were believed to improve lactation, promote healing, and remove toxins. Toor daal (Hindi) with rice was consumed by 99 (99.0%) participants as it was considered nutritious and easily digestible. Jeera (Hindi)/fenugreek porridge and herbal/Ayurvedic preparations were consumed by 73 (73.0%) women each for relief of backache, improvement of breast milk secretion, and enhancement of postpartum recovery. Milk consumption was reported by 54 (54.0%) participants, while beetroot and yam preparations were consumed by 39 (39.0%) and 42 (42.0%) women, respectively, for perceived improvement in blood production and wound healing (Table 5).

**Table 5:** Commonly preferred foods during the postnatal period and perceived benefits (n=100). Data are presented as n (%). The table presents the commonly preferred foods and traditional preparations consumed during the postpartum period, together with their perceived health benefits according to cultural beliefs.

Preferred food/preparation	n (%)	Perceived cultural benefit/reason
“Hot foods” (pepper/garlic/onion preparations)	87 (87)	Believed to improve lactation, healing, and toxin removal
Toor daal (Hindi) with rice	99 (99)	Considered nutritious and easily digestible
Jeera (Hindi)/fenugreek porridge	73 (73)	Believed to relieve backache and improve breast milk secretion
Herbal/Ayurvedic preparations “kashayas”	73 (73)	Believed to enhance postpartum recovery and prevent infections
Milk consumption	54 (54)	Believed to improve maternal strength and lactation
Beetroot preparations	39 (39)	Consumed for “blood production”
Yam preparations	42 (42)	Believed to improve wound healing
Banana stem/banana flower preparations	28 (28)	Believed to remove toxins and improve uterine cleansing

Association between religion and selected cultural practices showed no statistically significant differences between Hindu and Muslim participants. Consumption of “hot foods” was reported by 82 (87.2%) Hindu women and five (83.3%) Muslim women (Fisher's exact test, p=0.812). Restriction in food quantity was practiced by 91 (96.8%) Hindu women and all Muslim women (six, 100%). Head covering was observed among 86 (91.5%) Hindu women and six (100%) Muslim women. Similarly, restriction of outdoor activity, reduced water intake, abdominal binding, and restriction of curd/buttermilk were comparably prevalent among both religious groups, with no statistically significant association observed (all p>0.05) (Table 6).

**Table 6:** Association between religion and selected cultural practices during the postnatal period. Data are presented as n (%). Associations were analyzed using Fisher's exact test because one or more expected cell frequencies were <5. A p<0.05 was considered statistically significant.

Cultural practice	Hindu (n=94)	Muslim (n=6)	Statistical test	p-Value
Consumption of "hot foods"	82 (87.2)	5 (83.3)	Fisher's exact test	0.812
Restriction in food quantity	91 (96.8)	6 (100)	Fisher's exact test	0.731
Reduced water intake	53 (56.4)	4 (66.7)	Fisher's exact test	0.624
Abdominal binding	53 (56.4)	3 (50)	Fisher's exact test	0.756
Head covering	86 (91.5)	6 (100)	Fisher's exact test	0.588
Restriction of curd/buttermilk	31 (33)	1 (16.7)	Fisher's exact test	0.411
Restriction of outdoor activity	81 (86.2)	5 (83.3)	Fisher's exact test	0.844

Regarding self-reported postpartum weight change, 46 (46.0%) women reported weight loss during the postpartum period, while 28 (28.0%) reported weight gain. No significant change in body weight was observed among 26 (26.0%) participants (Table 7). Associations between selected variables and self-reported postpartum weight change were evaluated using appropriate statistical tests. Consumption of “hot foods” demonstrated a statistically significant association with self-reported postpartum weight change (p=0.011). Although weight loss was more frequently reported among primiparous women and women who underwent LSCS, these associations were not statistically significant (p>0.05). Likewise, no statistically significant association was observed between religion, restriction in quantity of food intake, or vegetable restriction and self-reported postpartum weight change (Table 8).

**Table 7:** Distribution of self-reported postpartum weight change among participants (n=100). Data are presented as n (%). The table describes the distribution of self-reported postpartum weight change among study participants, categorized as weight loss, weight gain, or no significant change.

Self-reported postpartum weight change	n (%)
Weight loss	46 (46)
Weight gain	28 (28)
No significant change	26 (26)

**Table 8:** Association between selected variables and self-reported postpartum weight change. Data are presented as n (%). Associations were analyzed using Pearson's chi-square test when assumptions were satisfied and Fisher's exact test when expected cell frequencies were <5. A p<0.05 was considered statistically significant. LSCS: lower segment cesarean section

Variables	Category (n)	Weight loss, n (%)	Weight gain, n (%)	No significant change, n (%)	Test and value	p-Value
Religion	Hindu (94)	43 (45.7)	26 (27.7)	25 (26.6)	Fisher's exact test	0.860
	Muslim (6)	3 (50)	2 (33.3)	1 (16.7)		
Parity	Primipara (53)	29 (54.7)	11 (20.8)	13 (24.5)	χ²=4.071	0.131
	Multipara (47)	17 (36.2)	17 (36.2)	13 (27.6)		
Mode of delivery	LSCS (55)	31 (56.4)	12 (21.8)	12 (21.8)	χ²=5.344	0.069
	Vaginal delivery (45)	15 (33.3)	16 (35.6)	14 (31.1)		
Restriction in quantity of food intake	Yes (97)	44 (45.4)	27 (27.8)	26 (26.8)	Fisher's exact test	0.570
	No (3)	2 (66.7)	1 (33.3)	0 (0)		
Consumption of “hot foods”	Yes (87)	44 (50.6)	20 (23)	23 (26.4)	Fisher's exact test	0.011
	No (13)	2 (15.4)	8 (61.5)	3 (23.1)		
Vegetable restriction	Yes (18)	11 (61.1)	4 (22.2)	3 (16.7)	Fisher's exact test	0.352
	No (82)	35 (42.7)	24 (29.3)	23 (28)		

## Discussion

The present study demonstrated that cultural beliefs and traditional postpartum practices remain highly prevalent among postnatal women. Restriction in the quantity of food intake was practiced by 97 (97.0%) participants, while consumption of “hot foods” was reported by 87 (87.0%) women. Similar findings were observed in the study conducted by Venkateswarlu et al., where traditional dietary modifications and food taboos were widely practiced during the postpartum period [6]. In the present study, restriction of curd and buttermilk was observed among 32 (32.0%) women and restriction of fruits among six (6.0%) women due to beliefs regarding “cold foods,” delayed recovery, and illness in the infant. Comparable findings were reported in studies from Andhra Pradesh and other regions of India, where yogurt, fruits, pulses, and vegetables were commonly restricted because of prevailing hot-cold food concepts and cultural myths [6,14].

Traditional postpartum confinement practices were also highly prevalent in the present study. Restriction of outdoor activity was observed among 86 (86.0%) women, reduced physical activity among 81 (81.0%), and head covering among 92 (92.0%) participants. Oil massage/body massage and consumption of home remedies were practiced by 74 (74.0%) and 73 (73.0%) women, respectively. Similar postpartum confinement practices have been documented in studies conducted in South India and Asian countries, where women commonly remained indoors for nearly 30-40 days after delivery and followed several traditional recovery rituals [6,14]. Venkateswarlu et al. reported that most women avoided outdoor activities and household work during the postpartum period, while Raven et al. and Liu et al. also described confinement practices as culturally accepted measures intended to protect the mother from weakness and infections [6,15,16].

The present study also observed widespread restriction of nutritious food items because of cultural beliefs. Eggs, fish, and mutton were restricted by eight (8.0%) women, while reduced water intake was practiced by 57 (57.0%) participants due to beliefs related to abdominal distension and delayed recovery. Similar observations were reported in qualitative studies on postpartum nutrition, where mothers avoided protein-rich foods, pulses, fruits, and water because these were believed to adversely affect maternal and infant health [6,13,14]. Bathula et al. reported that many postpartum women followed strict dietary restrictions during the initial weeks after delivery and commonly avoided fruits and protein-rich foods due to fear of illness and digestive disturbances [17]. Kudumula et al. similarly documented that almost all lactating mothers practiced one or more dietary restrictions during the postpartum period, particularly avoiding yogurt, fruits, leafy vegetables, eggs, and pulses [14].

In the present study, consumption of “hot foods” was the only factor that demonstrated a statistically significant association with self-reported postpartum weight change. Although weight loss was more frequently reported among primiparous women and women who underwent lower segment cesarean section (LSCS), these associations were not statistically significant. Similar observations regarding culturally perceived recovery-promoting foods have been reported in Indian and Asian studies, where traditional preparations containing pepper, garlic, and ginger were believed to enhance lactation, improve digestion, and promote maternal recovery [7,18,19]. However, because the present study employed a cross-sectional design and assessed self-reported postpartum weight change, the observed association should be interpreted cautiously and does not imply a causal relationship. Although several cultural practices may provide emotional support and promote family care, inappropriate dietary restrictions and reduced dietary diversity may adversely affect maternal nutritional status and postpartum well-being.

Strengths and limitations

The present study provides important insight into culturally influenced postpartum dietary practices, lifestyle modifications, and traditional beliefs among postnatal women attending a tertiary care hospital. Inclusion of multiple domains such as food restrictions, preferred foods, confinement practices, and self-reported postpartum weight change adds comprehensiveness to the study. The study also highlights prevailing myths and culturally shared practices relevant to maternal nutrition and recovery in the Indian setting. However, the study was limited by its cross-sectional design and single-center setting, which may limit generalizability of findings. Self-reported postpartum weight change and dietary practices may also be subject to recall bias, reporting bias, and social desirability bias. Furthermore, the study did not adjust for potential confounding variables such as baseline nutritional status, gestational weight gain, or dietary intake, and the cross-sectional design precludes establishing temporal or causal relationships between cultural practices and postpartum weight change. In addition, detailed nutritional assessment and long-term maternal outcomes were not evaluated.

## Conclusions

The present study showed that cultural beliefs and traditional postpartum practices remain highly prevalent among postnatal women attending a tertiary care hospital. Dietary restrictions, consumption of culturally perceived “hot foods,” confinement practices, reduced physical activity, and restriction of certain nutritious foods were commonly followed during the postpartum period. Consumption of culturally perceived “hot foods” was significantly associated with self-reported postpartum weight change, whereas no significant associations were observed with parity or mode of delivery. Although several traditional practices were culturally accepted and widely practiced, some dietary restrictions and lifestyle modifications may influence perceived postpartum recovery and maternal nutritional well-being; however, these findings should be interpreted with caution because of the cross-sectional study design and the use of self-reported outcome measures. Future multicenter and longitudinal studies with objective nutritional assessment and direct measurement of postpartum weight changes are required to further evaluate the impact of these practices on maternal health outcomes.

## Appendices

**Table 9:** Semi-structured questionnaire used for data collection.

Section	Variables/questions	Response options
Participant details	Case record number	__________
	Date	___/___/______
	Age (years)	__________
	Religion	□ Hindu □ Muslim □ Others (specify: __________)
Obstetric details	Gravida	__________
	Para	__________
	Postnatal day	__________
	Mode of delivery	□ LSCS □ Vaginal delivery
Section A. Dietary practices during the postpartum period	1. Restriction in quantity of food intake	□ Yes □ No
	2. Consumption of "hot foods" (pepper/garlic/onion preparations)	□ Yes □ No
	3. Consumption of vegetables	□ Yes □ No
	4. Restriction of curd/buttermilk	□ Yes □ No
	5. Restriction of brinjal/potato/pumpkin	□ Yes □ No
	6. Restriction of fruits	□ Yes □ No
	7. Restriction of protein-rich foods (eggs/fish/mutton)	□ Yes □ No
	8. Consumption of toor dal (Hindi) with rice	□ Yes □ No
	9. Daily water intake	□ <2 L/day □ ≥2 L/day
Section B. Traditional postpartum practices	10. Head covering practice	□ Yes □ No
	11. Restriction of outdoor activity/confinement at home	□ Yes □ No
	12. Reduced physical activity	□ Yes □ No
	13. Oil massage/body massage	□ Yes □ No
	14. Consumption of home remedies/Ayurvedic preparations	□ Yes □ No
	15. Abdominal binding	□ Yes □ No
	16. Wearing warm clothing irrespective of climate	□ Yes □ No
Section C. Commonly restricted foods and associated cultural beliefs	Curd/buttermilk	□ Yes □ No Reason/belief: __________________
	Brinjal	□ Yes □ No Reason/belief: __________________
	Potato/pumpkin	□ Yes □ No Reason/belief: __________________
	Fruits	□ Yes □ No Reason/belief: __________________
	Eggs/fish/mutton	□ Yes □ No Reason/belief: __________________
	Sesame seeds	□ Yes □ No Reason/belief: __________________
	Excess water intake	□ Yes □ No Reason/belief: __________________
Section D. Commonly preferred foods and perceived benefits	"Hot foods" preparations	□ Yes □ No Perceived benefit: __________________
	Toor dal (Hindi) with rice	□ Yes □ No Perceived benefit: __________________
	Jeera (Hindi)/fenugreek porridge	□ Yes □ No Perceived benefit: __________________
	Herbal/Ayurvedic preparations	□ Yes □ No Perceived benefit: __________________
	Milk consumption	□ Yes □ No Perceived benefit: __________________
	Beetroot preparations	□ Yes □ No Perceived benefit: __________________
	Yam preparations	□ Yes □ No Perceived benefit: __________________
	Banana stem/banana flower preparations	□ Yes □ No Perceived benefit: __________________
Section E. Outcome assessment	17. Self-reported postpartum weight change	□ Weight loss □ Weight gain □ No significant change
Investigator details	Participant signature/thumb impression	__________________
	Investigator signature	__________________
